# Chemistry of the Interaction and Retention of Tc^VII^ and Tc^IV^ Species at the Fe_3_O_4_(001) Surface

**DOI:** 10.1021/acs.jpcc.3c00688

**Published:** 2023-04-12

**Authors:** Enrico Bianchetti, Augusto F. Oliveira, Andreas C. Scheinost, Cristiana Di Valentin, Gotthard Seifert

**Affiliations:** † Dipartimento di Scienza dei Materiali, Università di Milano Bicocca, Via Roberto Cozzi 55, 20125 Milano, Italy; ‡ Institute of Resource Ecology, Helmholtz-Zentrum Dresden-Rossendorf (HZDR), Forschungsstelle Leipzig, Permoserstr. 15, 04318 Leipzig, Germany; § Theoretische Chemie, Technische Universität Dresden, Bergstr. 66c, 01062 Dresden, Germany; ∥ Institute of Resource Ecology, Helmholtz-Zentrum Dresden-Rossendorf (HZDR), Bautzner Landstr. 400, 01328 Dresden, Germany; ⊥ The Rossendorf Beamline (ROBL) European Synchrotron Radiation Facility (ESRF), Avenue des Martyrs 71, 38043 Grenoble, France; # BioNanoMedicine Center NANOMIB, Università di Milano Bicocca, Via Raoul Follereau 3, 20900 Monza, Italy

## Abstract

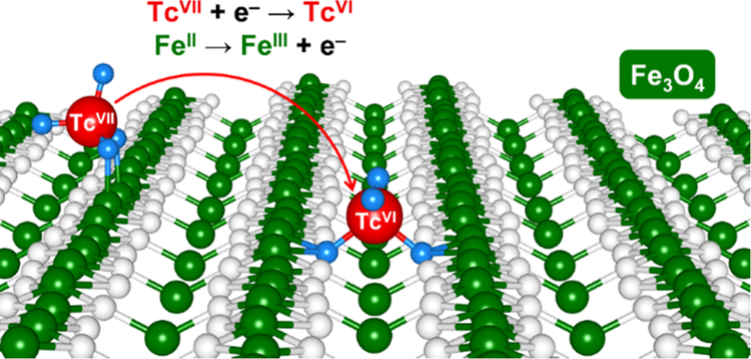

The pertechnetate ion Tc^VII^O_4_^–^ is a nuclear fission product whose major issue is the high mobility in the environment. Experimentally, it is well known that Fe_3_O_4_ can reduce Tc^VII^O_4_^–^ to Tc^IV^ species and retain such products quickly and completely, but the exact nature of the redox process and products is not completely understood. Therefore, we investigated the chemistry of Tc^VII^O_4_^–^ and Tc^IV^ species at the Fe_3_O_4_(001) surface through a hybrid DFT functional (HSE06) method. We studied a possible initiation step of the Tc^VII^ reduction process. The interaction of the Tc^VII^O_4_^–^ ion with the magnetite surface leads to the formation of a reduced Tc^VI^ species without any change in the Tc coordination sphere through an electron transfer that is favored by the magnetite surfaces with a higher Fe^II^ content. Furthermore, we explored various model structures for the immobilized Tc^IV^ final products. Tc^IV^ can be incorporated into a subsurface octahedral site or adsorbed on the surface in the form of Tc^IV^O_2_·*x*H_2_O chains. We propose and discuss three model structures for the adsorbed Tc^IV^O_2_·2H_2_O chains in terms of relative energies and simulated EXAFS spectra. Our results suggest that the periodicity of the Fe_3_O_4_(001) surface matches that of the TcO_2_·2H_2_O chains. The EXAFS analysis suggests that, in experiments, TcO_2_·*x*H_2_O chains were probably not formed as an inner-shell adsorption complex with the Fe_3_O_4_(001) surface.

## 1 Introduction

Technetium is a major concern due to its radiotoxicity, high fission yield in nuclear reactors, long half-life, and long mobility in the environment. The β-emitting ^99^Tc isotope is especially concerning. With a formation yield of ca. 6% in both ^235^U and ^239^Pu nuclear reactors and a half-life of ca. 2.1 × 10^5^ years,^1^^99^Tc will be the main radiation emitter 10^4^–10^6^ years after the production of the nuclear fuel waste.

In the absence of complexing agents besides oxygen and water, technetium assumes VII and IV oxidation states.^2^ In oxidizing conditions, Tc^VII^ is preferred and forms the pertechnetate ion (Tc^VII^O_4_^–^), which is highly soluble and mobile in the environment due to its weak interaction with mineral surfaces.^3^ On the other hand, in nonoxidizing conditions, technetium is reduced to Tc^IV^, precipitating as Tc^IV^O_2_·*x*H_2_O or forming adsorption complexes with mineral phases containing Fe^II^, which participate in the Tc^VII^ reduction.^4,5^

Magnetite (Fe^II^Fe_2_^III^O_4_) plays an important role in the immobilization of technetium in nuclear waste. In a typical geological nuclear waste repository, the spent nuclear fuel is enclosed in steel containers, which are then deposited in stable geological sites hundreds of meters below the surface; once full, the repository is sealed with bentonite clay and cement.^6^ In such an environment, magnetite forms as one of the main products of the anoxic corrosion of steel containers.^7^ It has been demonstrated that Fe^II^ in solid phases can quickly reduce Tc^VII^O_4_^–^ to Tc^IV^ species,^4,5^ whereas Fe^III^ solid phases can adsorb and incorporate Tc^IV^,^2,8^ hence the importance of magnetite in preventing the diffusion of Tc into the environment. It has been shown that Tc^IV^ remains adsorbed on or incorporated in the oxidized magnetite.^2,8^ However, the exact structure of the redox products has not been completely elucidated and is affected by several factors, such as pH, initial Tc concentration, and redox conditions of the aqueous phase, among others.

In 2016, Yalçintaş et al.,^9^ based on X-ray absorption near edge spectroscopy (XANES) and extended X-ray absorption fine structure (EXAFS) measurements, found that the end product of TcO_4_^–^ reduction by magnetite is related to the initial Tc content in solution, with higher concentrations (2 × 10^–4^ M) favoring adsorption of dimeric Tc^IV^ oxides onto the magnetite surface and lower concentrations (2 × 10^–5^ M) favoring incorporation of Tc^IV^ into the magnetite lattice. A gradual transition from exclusively adsorbed to exclusively incorporated Tc was also observed with decreasing Tc concentration. On the other hand, when using mackinawite (FeS) instead of magnetite, Yalçintaş et al.^9^ obtained noncrystalline TcO_2_·*x*H_2_O precipitates, for which two distinct linear chains of edge-sharing TcO_6_ octahedra with the H_2_O groups at the *trans* positions could be fitted to the EXAFS spectra; in the first structure, the Tc atoms are equally spaced along the chains (as proposed by Lukens et al.),^10^ whereas in the second, the Tc–Tc distances alternate between shorter and longer values, as in the TcO_2_ crystal structure.^11^ In 2022, Oliveira et al. ^12^ used density functional theory (DFT) calculations and EXAFS data to show that the precipitates are more likely formed by zigzag chains with terminal H_2_O at *cis* positions. Thus, it is clear that the interpretation of EXAFS spectra for these Tc systems is rather complex and can benefit from the aid of quantum chemical calculations.

In this work, we use a hybrid DFT functional method to explore the interaction of various Tc species with magnetite, starting from the adsorption of Tc^VII^O_4_^–^ onto the Fe_3_O_4_ (001) surface and proceeding with possible products of the full Tc^VII^O_4_^–^ reduction, namely, Tc^IV^ incorporated into the magnetite lattice and adsorbed Tc^IV^O_2_·2H_2_O chains. The Tc^VII^O_4_^–^ adsorption is analyzed in terms of spin densities, charges, and electronic density of states, whereas the structures of the Tc^IV^ species are discussed in terms of relative energies and simulated EXAFS spectra.

## 2 Methods and Models

### 2.1 Computational Methods

All DFT calculations were carried out with the HSE06^13,14^ hybrid exchange–correlation functional using the CRYSTAL17 package.^15,16^ This methodology has been shown to give a good description of the structural, electronic, and magnetic properties of magnetite systems.^17^ The Kohn–Sham orbitals were expanded in Gaussian-type orbitals: the all-electron basis sets are H|511G(p1), O|8411G(d1), Fe|86411G(d41), and Tc|976311(d631f1) according to the scheme previously used for Fe_3_O_4_.^17−20^ The irreducible Brillouin zone was sampled with a 3 × 3 × 1 *k*-point grid generated with the Monkhorst–Pack scheme.^21^ The convergence criterion of 4.5 × 10^–4^ hartree/bohr for atomic force was used during geometry optimization, and the convergence criterion for total energy was set to 10^–6^ hartree for all of the calculations. All structures (see details below) were constructed in such a way as to keep inversion symmetry (e.g., by adding adsorbate molecules in specific locations above and below the slab models) in order to minimize the appearance of artificial dipole moments.

The EXAFS spectra were simulated for optimized structures with FEFF9.6.4^22−24^ using the self-consistent field mode with a global Debye–Waller factor of 0.003 Å, amplitude reduction factor of 0.9, and Δ*E*_0_ = 0.

### 2.2 Models of the Fe_3_O_4_(001) Surface

The (001) termination is one of the most stable magnetite surfaces.^25,26^ Under the alkaline conditions of geological repositories,^8^ it is expected to be one of the most exposed surfaces in nanostructures,^27,28^ according to the Wulff construction.^29^ For these reasons, we have used this surface in our model. In the [001] direction, the Fe_3_O_4_ consists of alternating planes containing tetrahedral iron (Fe_Tet_) atoms and octahedral iron (Fe_Oct_) coordinated to oxygen atoms. The most recent and reliable models for the (001) termination are based on a bulk truncation at the Fe_Oct_ and O plane. The distorted bulk truncation (DBT) model consists of a simple bulk truncation,^30^ whereas the subsurface cation vacancy (SCV) model shows a reconstruction that consists of an extra interstitial Fe_Tet_ atom in the second layer replacing two Fe_Oct_ atoms from the third layer (per the (√2 × √2)R45° unit cell).^31^ The DBT and SCV models for the Fe_3_O_4_(001) surface are shown in Figure S1 in the Supporting Information. Their relative stability is highly dependent on the concentration of adsorbing molecules in the environment: an increasing amount of carboxylic acid or water molecules adsorbed onto the surface is found to favor the DBT structure.^18,32−34^ In this work, both models were constructed as a (1 × 1) 17-layer slab with inversion symmetry, as previously done by some of us.^18,35^ The five layers in the middle of the slab were kept fixed at the bulk positions, whereas the other layers were free to relax.

### 2.3 Models for the Adsorption of TcO_4_^*n*–^ onto the Fe_3_O_4_(001) Surface

Different complexes were constructed by adsorbing or embedding TcO_4_^*n*–^ into different sites of the DBT and SCV surface models. The SCV surface being more oxidized (fewer Fe^II^ centers) than the DBT one, it is interesting to compare the reducing power of both surface models. Since the DBT and SCV surfaces have identical terminating layers, exposing four penta-coordinated Fe_Oct_ atoms per unit cell, the models were built with the same criteria. The coordination shell of the superficial undercoordinated Fe_Oct_ atoms was saturated with either molecular or dissociated water, based on experimental and computational results.^18,35,36^ To balance the total charge, the most reactive superficial oxygen atoms were decorated with a proper number of hydrogen atoms.^37−39^ All structures were optimized, and, for each surface, the two lowest energy structures were selected for further analysis. Here, only the models associated to the two lowest energy structures are described in detail. The first model was built by adsorbing a TcO_4_ species onto two superficial undercoordinated Fe_Oct_ atoms, a H_2_O molecule, and an OH group onto the two remaining superficial undercoordinated Fe_Oct_ atoms. Two superficial oxygen atoms were decorated with two hydrogen atoms. The second model was built by attaching a TcO_2_ fragment to two superficial oxygen atoms, forming a TcO_4_ species embedded into the surface. The four superficial undercoordinated Fe_Oct_ atoms were saturated by one H_2_O molecule and three OH groups. No superficial oxygen atoms were decorated with hydrogen atoms. These two models have the same number of atoms for each element.

### 2.4 Models for the Incorporation of Tc^IV^ into the Fe_3_O_4_(001) Surface

Four models of Tc^IV^ incorporation were considered, one based on the SCV surface and three on the DBT surface. In all of them, an Fe_Oct_ atom from the third layer was replaced by a Tc atom. In two of the DBT-based models, an Fe vacancy was created in addition to the Tc-for-Fe substitution (i.e., two Fe_Oct_ atoms were replaced with one Tc and one vacancy). Several DBT-based models were created with the Fe vacancy at different positions with respect to the Tc atom, but only the two structures with the lowest energies were selected for further analysis. We considered both SCV and DBT surface models because (i) the SCV, being more oxidized than the DBT and characterized by the presence of iron vacancies, bears stronger resemblance to maghemite, which is expected to be one of the main final products of the magnetite oxidation by TcO_4_^–^, and (ii) recent experimental and theoretical findings show that under certain circumstances, the diffusion of other transition-metal atoms could reverse the SCV reconstruction, restoring a DBT surface, which presents the diffusing transition-metal atom (Tc in this case) instead of Fe occupying an octahedral site in the third layer.^40−42^

### 2.5 Models for the Adsorption of TcO_2_·2H_2_O Chains onto the Fe_3_O_4_(001) Surface

Three models were considered for the TcO_2_·2H_2_O infinite chains, based on the work by Oliveira et al.^12^ Each chain consists of edge-sharing TcO_6_ octahedra with terminal H_2_O groups occupying two corner positions. In α-TcO_2_·2H_2_O, the TcO_6_ octahedra form a linear chain with the terminal H_2_O in *trans* configuration and Tc–Tc nearest neighbors alternating longer and shorter distances along the chain as in the TcO_2_ (**P**2_1_/*c*) crystal structure.^11^ In β-TcO_2_·2H_2_O, the TcO_6_ octahedra form a zigzag chain, similar to ReO_2_ (*Pbcn*)^43^—note that Re is regarded as a Tc analogue—with the H_2_O groups at *cis* positions and identical distances for the Tc–Tc nearest neighbors. The last model, γ-TcO_2_·2H_2_O, differs from α-TcO_2_·2H_2_O for having identical Tc–Tc nearest distances along the chain, as in ReO_2_ (*P*4_2_/*mnm*).^44^ Oliveira et al.^12^ found β-TcO_2_·H_2_O to be the most energetically favored structure, with γ-TcO_2_·2H_2_O being the least favored.

The adsorption complexes were constructed by removing the H_2_O groups from one side of the TcO_2_·2H_2_O chains and placing the resulting structures on the Fe_3_O_4_(001) bare surface at bonding distance. Different models were constructed for each chain to explore different orientations on the surface. All structures were optimized, and the lowest energy structure of adsorbed α, β, and γ chains was used for further analysis. The investigation was restricted to the SCV surface because, being more oxidized than the DBT and characterized by the presence of iron vacancies, it bears stronger resemblance to maghemite, which is expected to be one of the main final products of the magnetite oxidation by TcO_4_^–^, as already discussed in Section 2.4. Furthermore, the SCV differs from the DBT only in the structure of the second and third layers and in the Fe^II^/Fe^III^ ratio, and it is reasonable to suppose that these differences do not influence the adsorption properties, especially when no redox reactions involve the Fe^II^/Fe^III^ pair, as in this case.

## 3 Results

### 3.1 Adsorption of TcO_4_^*n*–^ onto the Fe_3_O_4_(001) Surface

**Figure 1:**
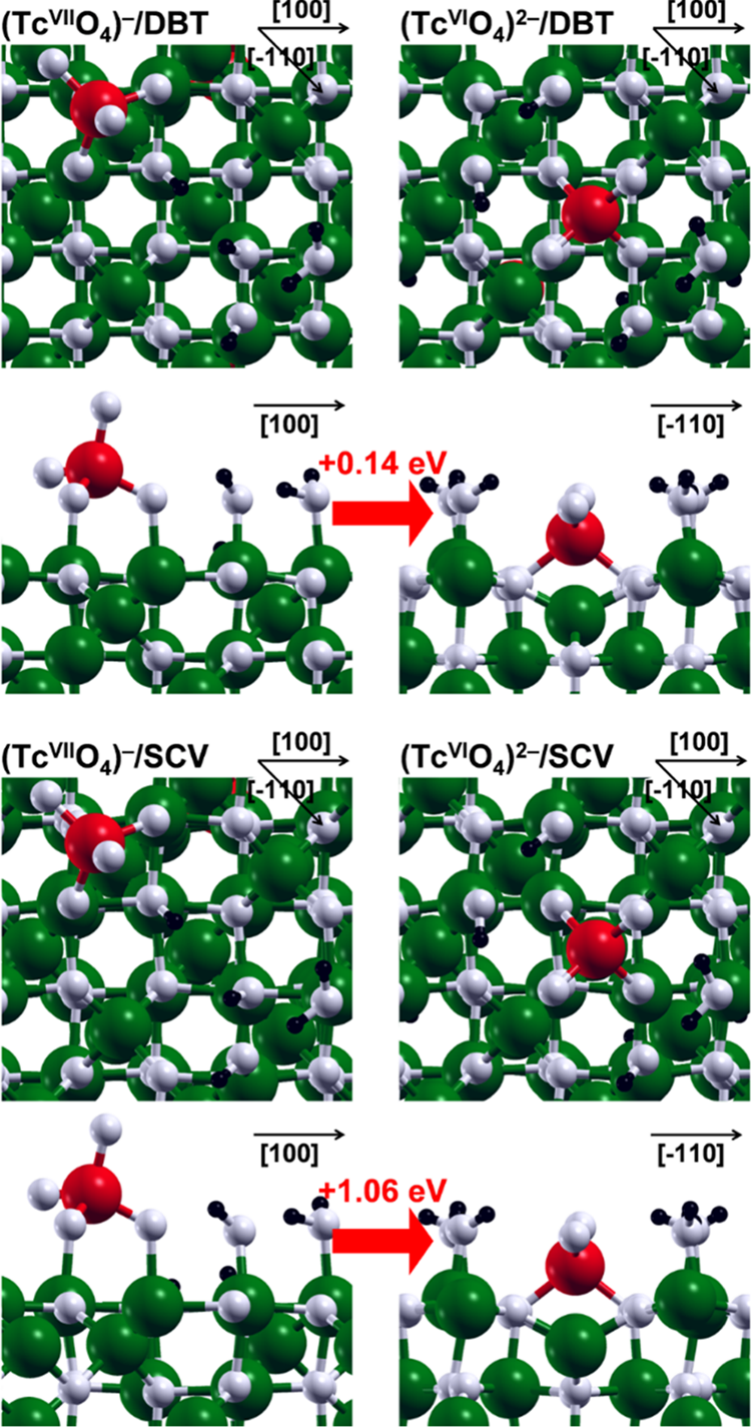
Top and side views of the optimized structures for the lowest-energy TcO_4_^*n*–^ complexes adsorbed onto the DBT (on the top) and SCV (on the bottom) Fe_3_O_4_(001) surfaces. The black, white, green, and red beads represent H, O, Fe, and Tc, respectively. The black arrows indicate the crystallographic directions.

In the first part of this study, we simulated the interaction of TcO_4_^*n*–^ species with the Fe_3_O_4_(001) surface by considering that the ions may either just adsorb by binding to undercoordinated surface Fe ions or become involved in surface reactivity leading to their surface embedding. The details of the models are described in the Methods and Models section. For both DBT and SCV surfaces, we have selected the two lowest-energy adsorption complexes, shown in Figure 1. In the models reported in the left panels of Figure 1 (referred to as (Tc^VII^O_4_)^−^/DBT and (Tc^VII^O_4_)^−^/SCV, as discussed below), TcO_4_^*n*–^ is adsorbed on two penta-coordinated Fe_Oct_ of the surface through two μ-O (i.e., twofold coordinated oxygen) bridging atoms. In the models shown in the right panels of Figure 1 (referred to as (Tc^VI^O_4_)^2–^/DBT and (Tc^VI^O_4_)^2–^/SCV, as discussed below), TcO_4_^*n*–^ becomes embedded in the surface through two μ_4_-O (i.e., fourfold coordinated oxygen) bridging atoms. This second adsorption site is the same that is generally preferred by single metal atoms adsorbed on the magnetite (001) surface, according to several recent studies.^40,45,46^

**Figure 2:**
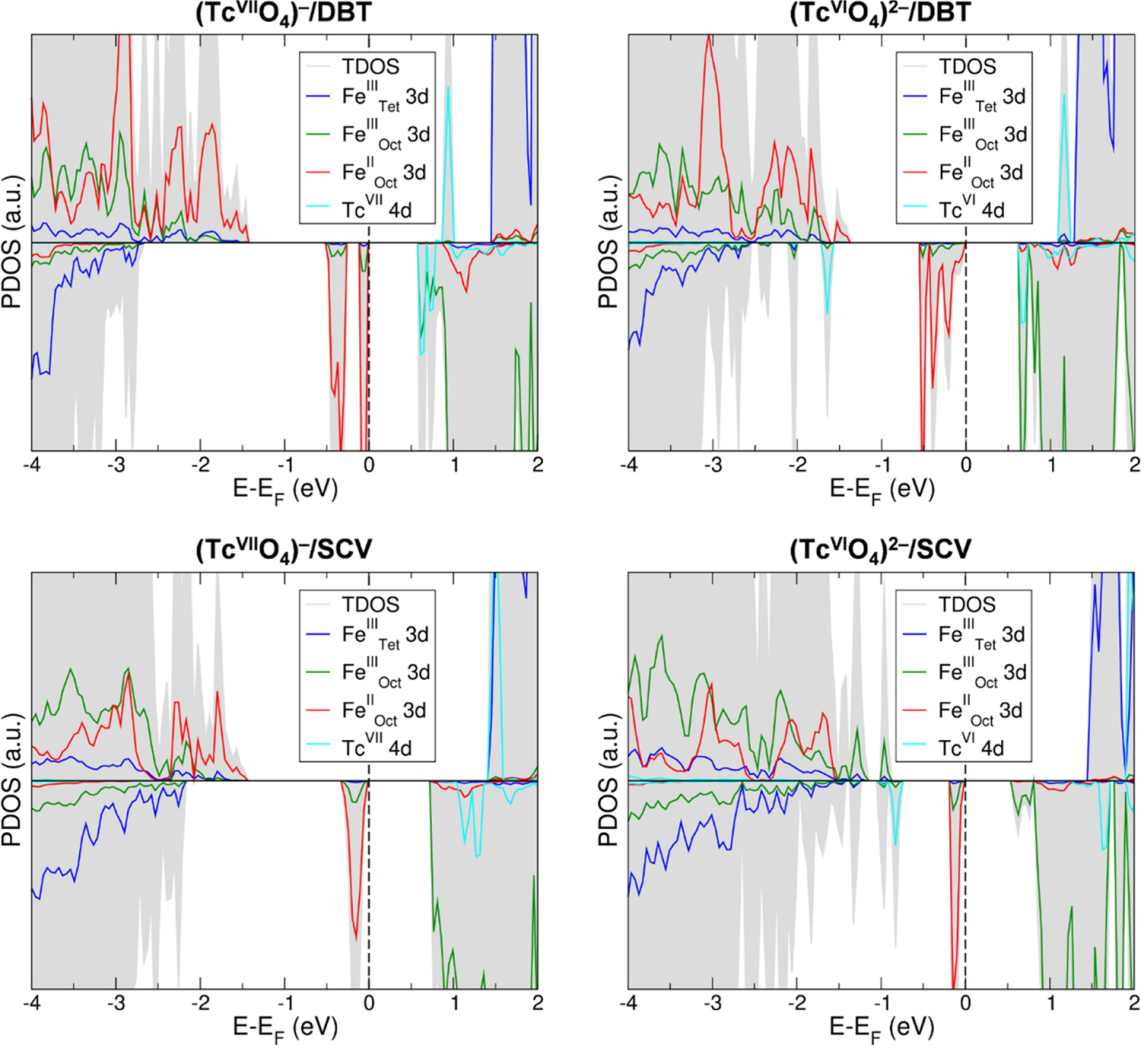
PDOS for the lowest-energy TcO_4_^*n*–^ complexes adsorbed onto the DBT (on the top) and SCV (on the bottom) Fe_3_O_4_(001) surfaces, shown in Figure 1.

The (Tc^VII^O_4_)^−^/DBT and (Tc^VII^O_4_)^−^/SCV models are characterized by the presence of technetium in its VII oxidation state. As we can see in the PDOS in Figure 2, there are no Tc 4d states (Figure 2, cyan curve) in the valence band. All technetium 4d orbitals are located in the conduction band. Furthermore, Tc is characterized by almost null spin polarization. These findings are compatible with a Tc^VII^ species, corresponding to the electronic configuration [Kr]. On the other hand, in (Tc^VI^O_4_)^2–^/DBT and (Tc^VI^O_4_)^2–^/SCV models, we observe that Tc^VII^ is reduced to Tc^VI^ while one Fe^II^ (in the fifth and in the seventh layer, respectively) of the magnetite surface is oxidized to Fe^III^. The reduction of Tc^VII^ to Tc^VI^ is in line with the Mulliken charge decrease of 5% and with a new Tc 4d contribution to the valence band in the spin-down channel of the PDOS (Figure 2, cyan curve). Indeed, the Mulliken spin density value of −0.7 μ_B_ for Tc in both (Tc^VI^O_4_)^2–^/DBT and (Tc^VI^O_4_)^2–^/SCV is consistent with a Tc^VI^ species with electronic configuration [Kr]4d^1^, i.e., with one unpaired electron. The Fe^II^ (high spin [Ar]3d^6^ configuration) oxidation to Fe^III^ (high spin [Ar]3d^5^ configuration) is confirmed by the Mulliken charge and spin density increase of 15% and from 3.7 to 4.2 μ_B_, respectively. Despite the similar structural properties of the DBT and SCV adsorption complexes, the redox energies differ considerably: the reaction energy per Tc atom is 1.06 eV for (Tc^VII^O_4_)^−^/SCV → (Tc^VI^O_4_)^2–^/SCV and 0.14 eV for (Tc^VII^O_4_)^−^/DBT → (Tc^VI^O_4_)^2–^/DBT. The Tc^VII^ reduction to Tc^VI^ is more favorable on the DBT surface than on the SCV surface. This may be mainly due to two reasons: first, the DBT surface presents a higher content of Fe^II^ ions in comparison to the more oxidized SCV one; second, the electron transfer from one Fe^II^ center to the Tc^VII^ ion implies the formation of an electric dipole which is smaller on the DBT than on the SCV surface, since the distances between the Tc^VII^ ion and the Fe^II^ centers involved in its reduction to Tc^VI^ are found to be about 6 and 8 Å, respectively.

The reduction of Tc^VII^ to Tc^VI^ by a *simple* electron transfer (from the magnetite surface to the technetium atom) is likely the first step of a *complex* redox process, which is known to proceed rapidly, producing Tc^IV^ end-members at slightly alkaline pH. The process involves the oxidation of Fe^II^ close to the Tc adsorbate, changing the Tc geometry from tetrahedral to octahedral^47^—similarly to what happens during the reduction of Mn^VII^O_4_^–^ to Mn^IV^O_2_, passing through Mn^VI^O_4_^2–^.^48^ Given the complexity of the process and the lack of more specific information regarding the chemical species involved, the simulation of the full Tc^VII^O_4_^–^ reduction is out of the scope of this work. Therefore, we restrict our study to hypothetical final products: incorporation of Tc^IV^ in the magnetite slab and formation of Tc^IV^O_2_·*x*H_2_O chains adsorbed on the magnetite surface.

### 3.2 Incorporation of Tc^IV^ into the Fe_3_O_4_(001) Surface

**Figure 3:**
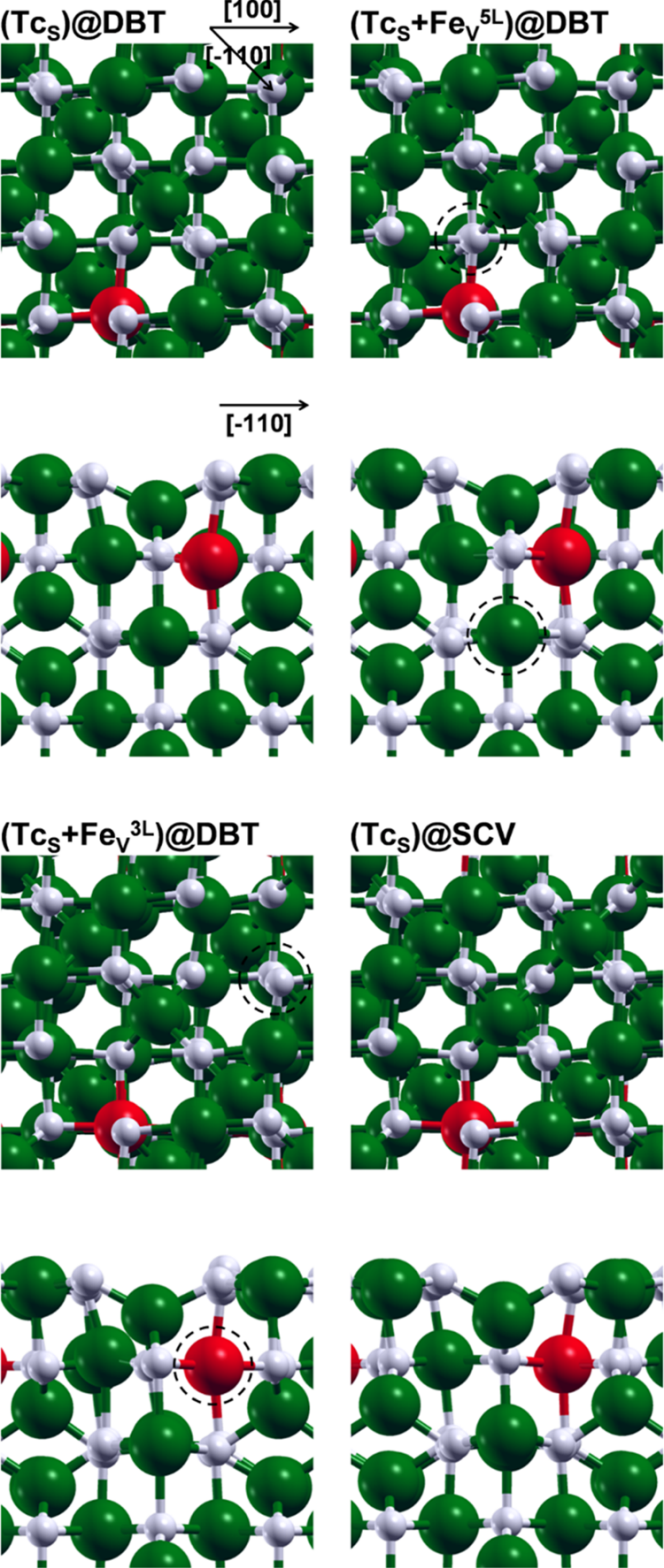
Top and side views of the optimized structures for the Tc^IV^ incorporation (Tc_S_), where we either only replaced a subsurface Fe_Oct_ in the third layer with a substitutional Tc atom (Tc_S_) ((Tc_S_)@DBT) or we also introduced a Fe_Oct_ vacancy (Fe_V_) ((Tc_S_ + Fe_V_^5L^)@DBT, (Tc_S_ + Fe_V_^3L^)@DBT, and (Tc_S_)@SCV). In the DBT cases, the dashed circle indicates the Fe_V_, whose location among the layers is given by the 3L (third layer) and 5L (fifth layer) apexes. The white, green, and red beads represent O, Fe, and Tc, respectively. The black arrows indicate the crystallographic directions.

In Figure 3, four models for the incorporation of Tc are shown, where we either only replaced a subsurface Fe_Oct_ in the third layer with a substitutional Tc atom (Tc_S_) ((Tc_S_)@DBT) or we also introduced a Fe_Oct_ vacancy (Fe_V_) ((Tc_S_ + Fe_V_^5L^)@DBT, (Tc_S_ + Fe_V_^3L^)@DBT, and (Tc_S_)@SCV). Note that SCV differs by one net Fe vacancy with respect to DBT, as detailed in Section 2.2.

The (Tc_S_)@DBT model presents a Tc in the IV oxidation state in place of a Fe^III^, as confirmed by the Mulliken charge value, which is lower than those found for Tc^VII^ and Tc^VI^, and almost identical to that obtained for Tc in the rutile phase of TcO_2_. The charge balance of the system is achieved by the reduction of a Fe^III^ ion to Fe^II^, indicated by the decrease of the Mulliken charge (15%) and spin density (from 4.2 to 3.7 μ_B_). This incorporation scheme consists of two Fe^III^ ions being replaced with a Tc^IV^-Fe^II^ pair, as already observed in previous computational studies investigating Tc incorporation in bulk hematite and magnetite.^49,50^

Similarly to (Tc_S_)@DBT, the (Tc_S_ + Fe_V_^5L^)@DBT, (Tc_S_ + Fe_V_^3L^)@DBT, and (Tc_S_)@SCV models also present Tc in the IV oxidation state. However, in this case, the Mulliken charges indicate that one Tc^IV^ ion replaces two Fe^II^ ions with respect to the pristine DBT surface, keeping the charge neutrality of the system, as previously observed for Tc-doped bulk magnetite.^50^ These models resemble what is observed in the oxidation process from magnetite (Fe_3_O_4_) to maghemite (γ-Fe_2_O_3_), which have the same structure, but the Fe^II^ ions in magnetite are replaced by Fe^III^ ions and vacancies in maghemite.^51^ Among the oxidized systems (namely, (Tc_S_ + Fe_V_^5L^)@DBT, (Tc_S_ + Fe_V_^3L^)@DBT, and (Tc_S_)@SCV), (Tc_S_)@SCV is the most stable one in terms of relative total energies in comparison to (Tc_S_ + Fe_V_^5L^)@DBT and (Tc_S_ + Fe_V_^3L^)@DBT (by +1.65 and +1.32 eV, respectively).

**Figure 4:**
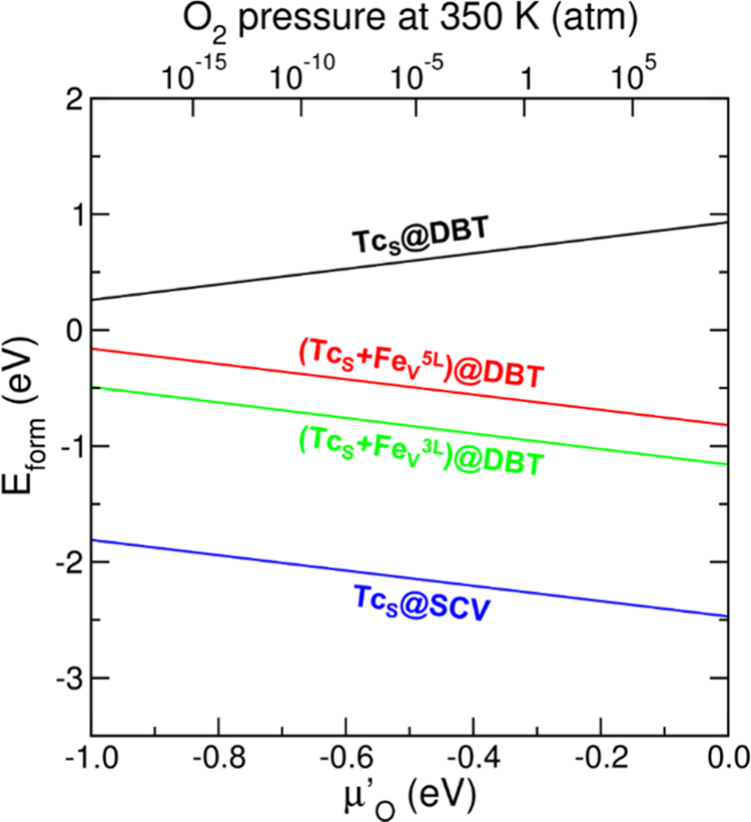
Formation energies (*E*_form_) as a function of oxygen chemical potential (μ_O_′, bottom *x*-axis) or as a function of oxygen pressure at the fixed temperature of 350 K (top *x*-axis) for Tc-incorporated models.

Furthermore, to compare the relative stability of the reduced ((Tc_S_)@DBT) and oxidized ((Tc_S_ + Fe_V_^5L^)@DBT, (Tc_S_ + Fe_V_^3L^)@DBT, and (Tc_S_)@SCV) systems, we plotted their formation energy (*E*_form_) as a function of oxygen chemical potential (μ_O_’) (see Figure 4), as detailed in Section S1 in the Supporting Information. As expected, under oxygen-rich conditions (μ_O_′ = 0), the oxidized systems are significantly more stable than the reduced (Tc_S_)@DBT system. Moving to oxygen-poor conditions (μ_O_′ = −1), (Tc_S_)@DBT is stabilized, whereas (Tc_S_ + Fe_V_^5L^)@DBT, (Tc_S_ + Fe_V_^3L^)@DBT, and (Tc_S_)@SCV become less stable. However, no stability inversion is observed down to experimentally feasible low O_2_ pressure (ca. 10^–20^ atm). In particular, (Tc_S_)@SCV is the most stable model at all considered values of oxygen chemical potential.

### 3.3 Adsorption of TcO_2_·2H_2_O Chains onto the Fe_3_O_4_(001) Surface

An alternative surface reactivity discussed in the literature would lead to the formation of hydrated Tc^IV^O_2_ dimers or chains on the magnetite surface.^8,9,52^ To study this possibility, we first investigated a free-standing TcO_2_·2H_2_O chain, as described in the Methods and Models section. The β-TcO_2_·2H_2_O chain was found to be the most stable chain, with Tc–Tc and Tc–O distances of ca. 2.4 and 1.9 Å, respectively. The α-TcO_2_·2H_2_O chain was found to be less stable by +758 meV per Tc atom (as reported in Table 1), with alternating Tc–Tc distances of ca. 2.2 and 3.3 Å. Consequently, also Tc–O distances present alternating values: 1.9 Å when O is bridging Tc–Tc at a smaller distance and 2.1 Å when bridging the Tc–Tc at a longer separation. The γ-TcO_2_·2H_2_O transformed to the α chain during the geometry optimization.

**Table 1:** Relative Total Energies Per Tc Atom (in meV) of the α-TcO_2_·2H_2_O, β-TcO_2_·2H_2_O, and γ-TcO_2_·2H_2_O Chains in Vacuum and Adsorbed onto the SCV Fe_3_O_4_(001) Surface

	α-TcO_2_·2H_2_O	β-TcO_2_·2H_2_O	γ-TcO_2_·2H_2_O
vacuum	+758	0	
adsorbed on SCV Fe_3_O_4_(001)	+16	+343	0

**Figure 5:**
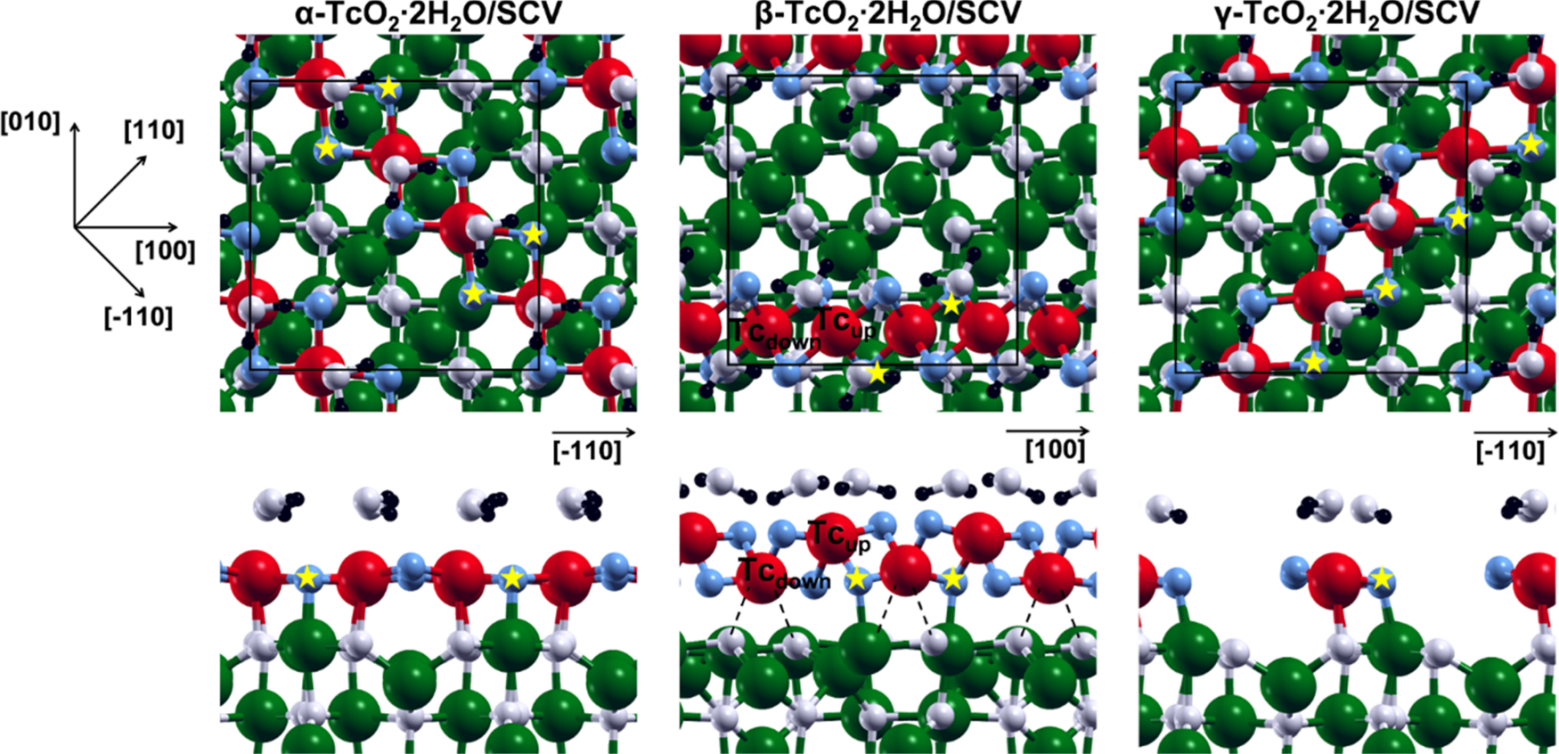
Top and side views of the optimized structures for the α-TcO_2_·2H_2_O, β-TcO_2_·2H_2_O, and γ-TcO_2_·2H_2_O (from left to right) chains adsorbed onto the SCV Fe_3_O_4_(001) surface. The axis orientation for all of the top views is shown on the left of the figure, whereas for the side views, it is shown in each single panel. The black, white, blue, green, and red beads represent H, O belonging to water molecules and magnetite, O belonging to TcO_2_, Fe, and Tc, respectively. The yellow stars indicate O from the chains interacting with surface Fe. Black dashed lines indicate weak Tc–O interactions.

As a next step, we investigated the interaction between the TcO_2_·2H_2_O chains with the magnetite surface. The periodicity of the magnetite surface and, in particular, of the alternating O–O distances along the [1̅10] direction, matches that of the α-TcO_2_·2H_2_O chain. The adsorbed α-TcO_2_·2H_2_O chain (Figure 5, α-TcO_2_·2H_2_O/SCV) presents only one kind of Tc (Figure 5, red beads) that is six-coordinated by four O from the chain itself (Figure 5, blue beads), one O shared with magnetite, and one O from a water molecule (Figure 5, white beads). Half of the O bridges in the chain (indicated with a yellow star in Figure 5) interacts with exposed undercoordinated Fe (Figure 5, green beads). The adsorption is driven by two types of interactions: one between Tc^IV^ and magnetite O, and the other between surface Fe^III^ and O belonging to TcO_2_·2H_2_O chains. α-TcO_2_·2H_2_O/SCV presents different alternating Tc–Tc distances with respect to the free-standing chain: ca. 2.8 and 3.1 Å versus 2.2 and 3.3 Å. This significant difference is due to the periodicity of the magnetite surface and, in particular, of the alternating O–O distances along the [1̅10] direction, i.e., the direction along which the α chain is adsorbed. Perpendicularly to the [1̅10] direction, the surface periodicity is significantly different. In particular, the periodicity of the almost constant O–O distances along the magnetite [110] direction matches that of the γ-TcO_2_·2H_2_O chain, which was not stable in vacuum. The adsorbed γ-TcO_2_·2H_2_O chain (Figure 5, γ-TcO_2_·2H_2_O/SCV) presents only one kind of Tc, whose coordination sphere is analogous to the one in α-TcO_2_·2H_2_O/SCV. Still, in analogy to α-TcO_2_·2H_2_O/SCV, half of the O atoms in the chain are interacting with superficial undercoordinated Fe. These structural similarities are translated into comparable energies: the total energy difference between the adsorbed γ and α chain is only 16 meV per Tc atom (as reported in Table 1), in favor of the former.

The free-standing β chain has a shorter lattice parameter than the α and γ chains due to its zigzag configuration. Consequently, it is not possible to efficiently adsorb the β-TcO_2_·2H_2_O along the diagonal direction of the cell as previously done for the α and γ one. Therefore, we studied the adsorption of the β-TcO_2_·2H_2_O along the [100] direction (Figure 5, β-TcO_2_·2H_2_O/SCV). In this case, two different kinds of Tc are present: one farther from the surface and one closer to it, labeled as Tc_up_ and Tc_down_ in Figure 5, respectively. Both Tc_up_ and Tc_down_ species are six-coordinated. Each Tc_up_ is coordinated by four O from the chain itself and by two O from two different water molecules, whereas each Tc_down_ is coordinated by four O from the chain itself and by two O shared with magnetite (Figure 5, dashed lines). Tc–Tc distances and other structural parameters of the adsorbed chain are not significantly different from what is observed for the free-standing chain. This configuration is found to be less favored than the α and γ one by +327 and +343 meV per Tc atom (see Table 1), respectively. This is an unexpected result, since the β-TcO_2_·2H_2_O chain is the most stable one in vacuum. This finding can be understood in terms of the (i) lower number (half compared to α and γ cases) of chain O atoms interacting with the magnetite surface and (ii) weaker Tc–O interactions (2.3–2.4 versus 1.9–2.0 Å for the α and γ chains) between the chain and the magnetite surface.

**Figure 6:**
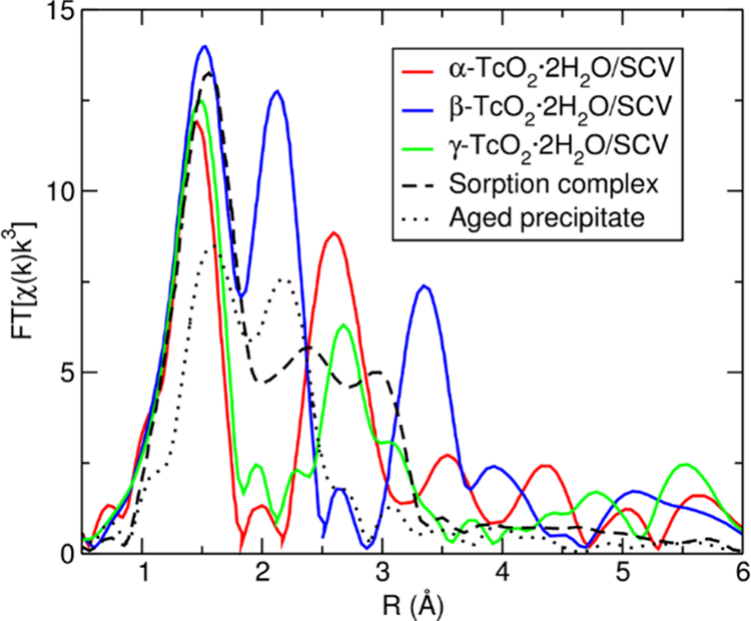
Experimental EXAFS spectra for the sorption complex by Yalçintaş et al.^9^ (black dashed line) and aged TcO_2_·*x*H_2_O precipitate by Oliveira et al.^12^ (black dotted line), and simulated EXAFS spectra for the α-TcO_2_·2H_2_O (red line), β-TcO_2_·2H_2_O (blue line), and γ-TcO_2_·2H_2_O (green line) chains adsorbed onto the SCV Fe_3_O_4_(001) surface.

Finally, we compared the experimental EXAFS spectra for the sorption complex by Yalçintaş et al.^9^ (Figure 6, black dashed curve) and for the aged TcO_2_·*x*H_2_O precipitate by Oliveira et al.^12^ (Figure 6, black dotted curve) with the calculated EXAFS spectra obtained for the simulated TcO_2_·2H_2_O chains adsorbed on the magnetite (001) surface just described. Regarding the experimental sorption complex curve, there is no match with the calculated curves of the simulated TcO_2_·2H_2_O chains models. We also modeled a magnetite/TcO_2_-dimer complex (shown in Figure S2 in the Supporting Information) in line with that suggested by Yalçintaş and collaborators.^9^ However, also in this case, the simulated EXAFS spectrum does not match the experimental one for the sorption complex. These results suggest that TcO_2_ chains (or dimers) are probably not formed as an inner-shell adsorption complex with the Fe_3_O_4_(001) surface, at least not immediately. Regarding the aged TcO_2_·*x*H_2_O precipitate curve, there are few similarities with the calculated β-TcO_2_·2H_2_O/SCV curve (Figure 6, blue curve). In particular, the positions of the first and second peaks are in fair agreement, as well as the presence of a small shoulder on the right of the second peak, but the third peak in the computed curve finds no correspondence in the experimental one. This result suggests that β-TcO_2_·2H_2_O chains might be formed in solution, not as an inner-shell adsorption complex with magnetite, and only afterward might precipitate and adsorb on the surface. Indeed, the formation of β-TcO_2_·2H_2_O chains is energetically favored over that of the α and γ ones in vacuum, not on the Fe_3_O_4_(001) surface. However, the agreement between the experimental aged precipitate curve and the computed β-TcO_2_·2H_2_O/SCV one is not good enough to definitively sustain this hypothesis. Therefore, the comparison between the experimental and the calculated data suggests the possibility that in the experiments, more radically modified and reconstructed Fe_3_O_4_(001) surfaces or even completely different surfaces, such as the (111) and (110), not considered in this work, might be involved.

## 4 Conclusions

In this work, based on a comprehensive hybrid DFT study, we investigated the chemistry of the interaction and retention of Tc^VII^ and Tc^IV^ species at the Fe_3_O_4_(001) surface.

As a first step, we studied the interaction and reactivity of the Tc^VII^O_4_^–^ ion with the magnetite surface. We suggest a possible initiation step for the reduction of Tc^VII^ to Tc^IV^ upon contact with the Fe_3_O_4_(001) surface. The adsorption of the Tc^VII^O_4_^–^ ion onto the magnetite surface leads to the formation of a reduced Tc^VI^ species without any change in the Tc coordination sphere through an electron transfer that is favored by the magnetite surfaces with a higher Fe^II^ content.

Furthermore, we explored various model structures for the possible final products of the full reduction from Tc^VII^ to Tc^IV^ at the Fe_3_O_4_(001) surface: Tc^IV^ incorporation or adsorption in the form of Tc^IV^O_2_·2H_2_O chains. The replacement of a Fe atom with a Tc atom in an octahedral site in the subsurface leads to the presence of an incorporated six-coordinated Tc^IV^, which is more stable in the SCV than in the DBT surface. Regarding the adsorption of TcO_2_·2H_2_O chains on magnetite, we propose three model structures that are characterized by three different symmetries. The periodicity of the Fe_3_O_4_(001) surface matches that of the TcO_2_·2H_2_O chains, and the adsorption is driven by two types of interactions: one between Tc^IV^ and magnetite O, and the other between surface Fe^III^ and O belonging to TcO_2_·2H_2_O chains. However, the comparison between the experimental and computed EXAFS spectra suggests that, in experiments, TcO_2_·*x*H_2_O chains were probably not formed as an inner-shell adsorption complex with the Fe_3_O_4_(001) surface.

To summarize, we have demonstrated that the Fe_3_O_4_(001) surface can adsorb and reduce Tc^VII^ complexes and retain Tc^IV^ species. In particular, we propose an initiation step for the reduction of Tc^VII^ and two retention mechanisms, i.e., Tc^IV^ ions incorporation into octahedral subsurface sites and adsorption in the form of TcO_2_·2H_2_O chains. Our results furnish a solid basis for any future study whose aim is to elucidate the steps of the complex reduction of Tc^VII^ to Tc^IV^ and, on the basis of the EXAFS analysis, could stimulate further investigations to understand whether the formation of TcO_2_·*x*H_2_O chains could take place in solution or even at other Fe_3_O_4_ surfaces.

## Supporting Information Available

Atomic structures of the DBT and SCV Fe_3_O_4_(001) surface models; optimized structure for the lowest-energy SCV Fe_3_O_4_(001)/TcO_2_-dimer complex; and protocol to evaluate the relative stability of Tc-incorporated models in terms of formation energy as a function of oxygen chemical potential (PDF)
