# Supplementary material for: Chemistry of the Interaction and Retention of TcVII and TcIV Species at the Fe3O4(001) Surface
Source: J Phys Chem C Nanomater Interfaces. 2023 Apr 12;127(16):7674–82. doi: 10.1021/acs.jpcc.3c00688 (PMC10150389; doi:10.1021/acs.jpcc.3c00688)
Supplement: Supplementary file 1 — jp3c00688_si_001.pdf [file jp3c00688_si_001.pdf]

Supporting Information

Chemistry of the Interaction and Retention of Tc^{VII} and Tc^{IV} Species at the Fe_3O_4(001) Surface

Enrico Bianchetti[1], Augusto F. Oliveira[2,3], Andreas C. Scheinost[4,5], Cristiana Di Valentin[1,6,*], and Gotthard Seifert[3,*]

[1] Dipartimento di Scienza dei Materiali, Università di Milano Bicocca, Via Roberto Cozzi 55, 20125 Milano, Italy

[2] Institute of Resource Ecology, Helmholtz-Zentrum Dresden-Rossendorf (HZDR), Forschungsstelle Leipzig, Permoserstr. 15, 04318 Leipzig, Germany

[3] Theoretische Chemie, Technische Universität Dresden, Bergstr. 66c, 01062 Dresden, Germany

[4] Institute of Resource Ecology, Helmholtz-Zentrum Dresden-Rossendorf (HZDR), Bautzner Landstr. 400, 01328 Dresden, Germany

[5] The Rossendorf Beamline (ROBL) European Synchrotron Radiation Facility (ESRF), Avenue des Martyrs 71, 38043 Grenoble, France

[6] BioNanoMedicine Center NANOMIB, Università di Milano Bicocca, Via Raoul Follereau 3, 20900 Monza, Italy

[*] Corresponding author: cristiana.divalentin@unimib.it, gotthard.seifert@tu-dresden.de

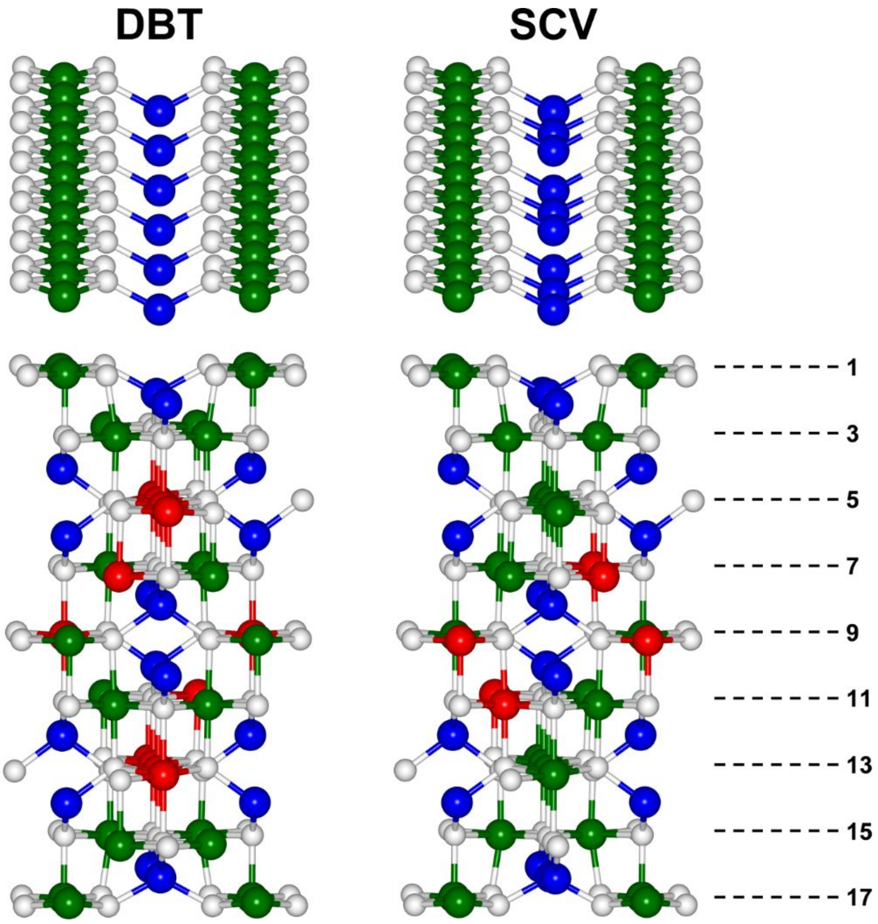

Figure S1. Top and side views of the atomic structures of the DBT and SCV $Fe_3O_4(001)$ surface models. The blue, green, red, and white beads represent $\mathrm{Fe}^{\mathrm{III}}_{\mathrm{Tet}}$, $\mathrm{Fe}^{\mathrm{III}}_{\mathrm{Oct}}$, $\mathrm{Fe}^{\mathrm{II}}_{\mathrm{Oct}}$, and $\mathrm{O}^{-\mathrm{II}}$, respectively. Layers are numbered on the right.

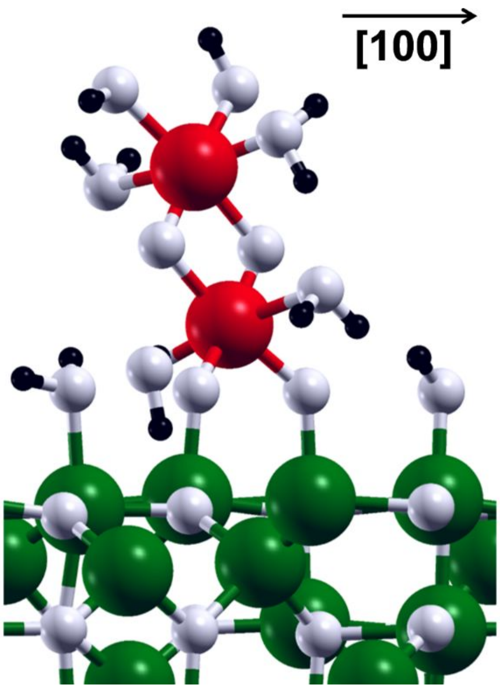

Figure S2. Side view of the optimized structure for the lowest-energy SCV Fe_3O_4(001)/TcO_2-dimer complex. Different SCV Fe_3O_4(001)/TcO_2-dimer models were constructed to explore different orientations and adsorption mode. All structures were optimized and only the lowest energy structure is shown here. The black, white, green, and red beads represent H, O, Fe, and Tc, respectively. The black arrow indicates the crystallographic direction.

S1. Formation energy against oxygen chemical potential

To examine the relative stability of Tc-incorporated systems, we compared their formation energies as a function of oxygen chemical potential, exploiting a protocol previously used by some of us.[1,2]

The formation energy (E_{form}) is computed as

$$E_{form} = E_{tot}(Tc_S) - [E_{tot}(DBT) + \mu_{Tc} - (1+n)\mu_{Fe}]$$

where $E_{tot}(Tc_S)$ and $E_{tot}(DBT)$ are the total energies of the Tc-incorporated systems (namely, (Tc_S)@DBT, $(Tc_S+Fe_V^{5L})$@DBT, $(Tc_S+Fe_V^{3L})$@DBT, and (Tc_S)@SCV) and pristine DBT surface, respectively, μ_{Tc} and μ_{Fe} are the chemical potential of Tc and Fe atoms, respectively, and n is the number of Fe vacancy.

The relative stability varies as a function of the oxygen chemical potential (μ_O), which is a parameter that characterizes the oxygen environment of the geological repositories. It is assumed that the system is in thermodynamic equilibrium with a reservoir of oxygen, which can give or take any amount of oxygen without changing its temperature and pressure.[3,4] Oxygen poor conditions correspond to low values of μ_O, whereas the oxygen-rich conditions correspond to high values of μ_O. The oxygen chemical potential μ_O is referred to the energy of an O atom in the O_2 molecule ($\mu_O = 1/2\mu_{O2} + \mu_O'$). The interval -1 eV ≤ μ_O' ≤ 0 was considered, where the value μ_O' = 0 corresponds to the oxygen-rich conditions when oxygen condensation occurs, whereas μ_O' = -1 eV corresponds to oxygen-poor conditions.

To define a source of Fe and Tc, it is assumed that the system is in thermodynamic equilibrium with a reservoir of bulk Fe_3O_4 and TcO_2 (in practice, this corresponds to assuming the Fe and Tc chemical potential to be $\mu_{Fe} = (\mu_{Fe3O4} - 4\mu_O)/3$ and $\mu_{Tc} = \mu_{TcO2} - 2\mu_O$, respectively), which can give or take any amount of Fe_3O_4 and TcO_2 without changing their temperature and pressure. Both bulk Fe_3O_4 and TcO_2 are stable in the interval -1 eV ≤ μ_O' ≤ 0. Indeed, μ_O' = -1 eV is less negative than one-quarter and one-half of the experimental enthalpy of formation of Fe_3O_4 (-11.6 eV[5]) and TcO_2 (-4.7 eV[6]), respectively.

The E_{form} of Tc-incorporated systems as a function of μ_O' are shown in Figure 4 in the main text. To translate the range of oxygen chemical potential into a more usual measure of oxygen concentration, the μ_O' values were converted to oxygen partial pressure (in atm) at the fixed temperature of 350 K (top x-axis), at which waste disposal is approximately assumed to occur.[7]

References

[1] Di Valentin, C.; Pacchioni, G.; Selloni, A. Theory of Carbon Doping of Titanium Dioxide. *Chem. Mater.* **2005**, *17*, 6656-6665.

[2] Kumaravel, V.; Bianchetti, E.; Mathew, S.; Hinder, S. J.; Bartlett, J.; Di Valentin, C.; Pillai, S. C. New Insights into Crystal Defects, Oxygen Vacancies, and Phase Transition of Ir-TiO_2. *J. Phys. Chem. C* **2021**, *125*, 23548-23560.

[3] Reuter, K.; Scheffler, M. Composition, Structure, and Stability of RuO_2(110) as a Function of Oxygen Pressure. *Phys. Rev. B* **2001**, *65*, 035406.

[4] Reuter, K.; Scheffler, M. Composition and Structure of the RuO_2(110) Surface in an O_2 and CO Environment: Implications for the Catalytic Formation of CO_2. *Phys. Rev. B* **2003**, *68*, 045407.

[5] Lide, D. R. *CRC Handbook of Chemistry and Physics*; CRC Press, 2010; Vol. 90.

[6] Rard, J. A.; Rand, M. H.; Anderegg, G.; Wanner, H. *Chemical Thermodynamics of Technetium*; Sandino, M. C. A., Osthols, E., Eds.; Amsterdam, 1999; Vol. 3.

[7] Madsen, F. T. Clay Mineralogical Investigations Related to Nuclear Waste Disposal. *Clay Miner.* **1998**, *33*, 109-129.
